# Exploring the Moderating Effect of Interpersonal Emotion Regulation Between the Integration of Opportunity and Resource and Entrepreneurial Performance

**DOI:** 10.3389/fpsyg.2021.756767

**Published:** 2021-10-28

**Authors:** Na Li, Yu Sun, Dake Jiang, Xiu Yang

**Affiliations:** ^1^School of Management, Changchun University, Changchun, China; ^2^School of Economics and Management, Dalian University of Technology, Dalian, China; ^3^Public Security Department, Criminal Investigation Police University of China, Shenyang, China; ^4^Accounting and Auditing College, Guangxi University of Finance and Economics, Nanning, China

**Keywords:** opportunity, resource, interpersonal emotion regulation, entrepreneurial performance, entrepreneurship

## Abstract

This study explains how start-ups obtain a high accumulated performance by aligning and employing an integration between opportunities and resources (IOR) in a dynamic environment and whether these potential benefits are associated with interpersonal emotion regulation. Using 274 enterprise samples, the findings confirm that an IOR has a significant positive effect on entrepreneurial performance. In addition, positive emotion and interpersonal relationship regulation positively moderate the relationship between IOR and entrepreneurial performance. This paper proposes a new concept of the IOR and measures it for the first time. Then, the relationship was explored between the IOR, interpersonal emotion regulation, and entrepreneurial performance. This research not only systematically integrates opportunities and resources and avoids their separation but also helps to reveal the context of entrepreneurship research, enrich entrepreneurship theory, and expand the boundaries.

## Introduction

Tremendous institutional and economic transitions are experienced by developing countries. Institutions and markets are highly uncertain in a transitional economy (Huang et al., 2015; Gao et al., 2018). Incomplete institutional structures and the unbalanced development of subsistence markets create tremendous risks for start-ups (Tan, 2001), although Chinese policy encourages the “masses to start their own businesses and to make innovations.” In particular, scarce resources are unfairly distributed due to the excessive interference of local administrations, and opportunities tend to be instantaneous due to the fuzzy boundary of subsistence markets and the fast pace of the competitive structure (Walder, 1995; Davcik and Sharma, 2016; Ge et al., 2018). This situation creates new challenges for start-up development: how can start-ups achieve high cumulative performance when coordinating opportunities and resources in a dynamic environment and are the potential benefits associated with interpersonal emotion regulation. The answers to these questions have substantial practical significance for assisting start-ups in effectively breaking through the restrictions of resource constraints and creating relevant opportunities to maximize their value in a dynamic and uncertain environment. It is highly valuable to explore the causal linkages between opportunities and resources in developing countries.

Opportunities and resources orientations in the dualistic theory of entrepreneurship are prominent in entrepreneurial research. In fact, opportunities and resources are mutually reinforcing and concomitant relations. However, the existing research mainly focuses on the single role of opportunities or resources and lacks the research from the perspective of integration (Teece, 2007; Sirmon et al., 2011). Further clarification is required to determine whether the complementary effect between opportunities and resources works well in a transitional economy and whether effective interpersonal emotion regulation promotes such efficiency. It is necessary to establish a theoretical model of integrating opportunities and resources from the perspective of system theory and to clarify its correlation with entrepreneurial performance.

Therefore, this study answers questions about how start-ups obtain a high accumulated performance by aligning opportunities and resources and employing integration between opportunities and resources (IOR) in a dynamic environment and whether these potential benefits are associated with interpersonal emotion regulation. This study is based on these ideas and guided by the maxims of the traditional Chinese philosophies of “the balance of yin and yang” and “the cultivation of both the internal and the external.” Therefore, first, we divide the IOR into two subdimensions, internal and external integration, and measure them. Ultimately, the IOR will also reflect their interaction and balance. Briefly, the paper explores the relationships between internal and external integration, the IOR, and entrepreneurial performance. Concurrently, we validate the moderating effects of positive emotion and interpersonal relationship regulation on the IOR and entrepreneurial performance.

Using 274 enterprise samples, we find that the entrepreneurial performance of start-ups in developing countries is optimal when internal and external integration are available at a collectively high level. Internal integration creates opportunities by piecing together existing resources. However, external integration is also required as opportunity discovery to cover shortages. That is, external integration has a complementary effect on internal integration. However, internal integration is essential to external integration. When faced with the resource constraints caused by fierce market competition, the auxiliary function of internal integration is necessary. In short, the IOR represents a high level of internal and external integration, and the two concepts can compensate for each other’s deficiencies so entrepreneurial efforts can perform highly in a transitional economy.

In addition, the results demonstrated that interpersonal emotion and relationship regulation further maximize the benefits of the IOR in social processes outside corporate boundaries. They also elucidated that interpersonal emotion regulation is not a negligible element in a transitional economy. For example, positive emotion regulation can enhance the cognitive flexibility of team members, stimulate them to discover further similarities or differences, and promote the integration of new and existing knowledge, which results in continuous competition for resources to the organization. In addition, interpersonal emotion regulation enhances team identity and trust by forming good interpersonal relationships between team members, thereby achieving cooperation and social promotion. Therefore, interpersonal emotion regulation can nurture incrementally fruitful performance benefits in the IOR.

This observation provides enlightenment for entrepreneurial start-ups in developing countries. First, the synergies of internal and external integration allow the returns of enterprises to outperform those in situations in which the two factors are separated. To ultimately capture such benefits, entrepreneurs must design strategies to significantly align internal and external integration. Second, entrepreneurial start-ups are inclined to effectively carry out interpersonal emotion regulation activities that can expand their gains in a turbulent system and a dynamic competitive market. Therefore, we encourage entrepreneurs to build positive team emotion and harmonious interpersonal relationships to obtain higher returns. This effort requires enterprises to pay attention to the training of identifying leaders’ tendencies and abilities in terms of interpersonal emotion regulation from the perspective of leaders’ development plans and improve and construct their effective strategies.

## Theoretical Background and Hypotheses

### Internal and External Integration, the IOR, and Entrepreneurial Performance

**Figure 1:**
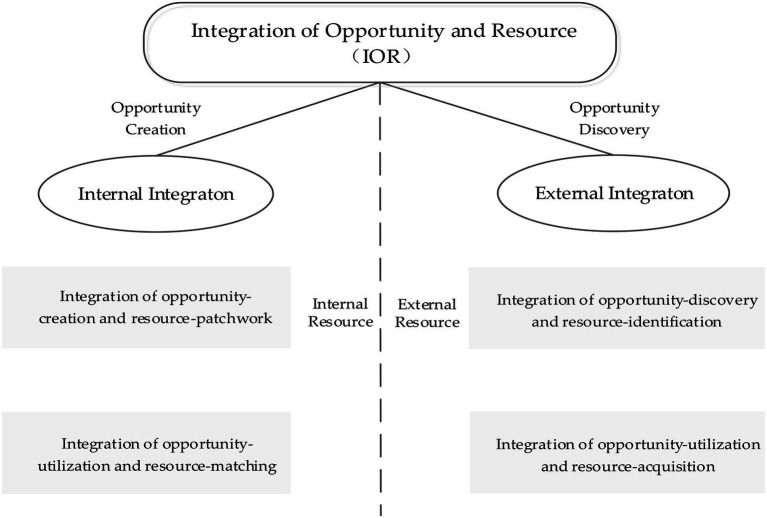
The model of the integration of opportunity and resource.

The core of entrepreneurial research is the matching of opportunities and resources. How to coordinate the two is the key to entrepreneurial success. Especially in the uncertain environment, it is more important to properly deal with the relationship between opportunities and resources. Only by comprehensively balancing opportunities and resources can achieve high performance. Leaning on either side of opportunities and resources will affect the effectiveness of entrepreneurial activities. Therefore, in the process of the interaction between opportunities and resources, the systematic integration of opportunities and resources has been advocated by scholars. This can not only avoid the one-sidedness of research, but also explore the essential process of entrepreneurship. On this basis, scholars further construct a theoretical system of opportunity and resource integration development behavior from the perspective of system theory and reveal the symbiotic evolutionary mechanism of the two. In recent years, the research results have gradually been enriched on the integrated exploitation of opportunities and resources. Clarifying the complex relationship between opportunities and resources and solving their “fragmented” situation are the origin of the IOR concept. This paper combines the conceptual design of the opportunity development process by Shane and Venkataraman (2000), the definition of resource development type by Brown et al. (2001), the systematic classification of the connotation between “opportunities under resources” and “resources under opportunities” by Ge et al. (2016), and the idea of merging opportunities and resources by Bhuian et al. (2005) and Boso et al. (2013). On these bases, this paper demarcates “internal and external resources” and combines the interaction between them into two parts, internal and external integration (see Figure 1).

First, the classification principle is based on the theory of *ying-yang balance*, a philosophy of dialectical materialism. The *ying-yang balance* shows that the existence and development of all things contain two factors that are opposite but reciprocal. The opposition between the yin and the yang is not absolute and static but relative and active. The *ying-yang balance* needs to be preserved and not neglected (Li, 2012). The *ying-yang balance* can direct the development of all things and is applicable to start-ups. In this paper, both internal and external integration are separated on account of two different resources, the internal and the external. Internal integration tends to elevate firms by creating opportunities according to “pieced-together internal resources” and capturing opportunities by “integrating the internal resources” of the moment, i.e., when opportunities are created within the firm and the resources used for creating these opportunities are derived from the firm (Lumpkin and Dess, 1996). External integration, however, emphasizes the decisive power outside the firm, especially the importance of identifying and acquiring external resources. When external integration works, firms tend to discover opportunities and utilize external resources. Therefore, internal and external integration are the two main modules of the entrepreneurial system. Although one is the yin and the other is the yang, they do not cross (Wiklund and Shepherd, 2003). However, only when they work together can they balance the entrepreneurial system, of which both are indispensable.

Second, internal and external integration are effective. Internal integration includes two subdimensions: identifying resources and opportunities and assigning resources while utilizing opportunities. The former represents the creation of opportunities by piecing together existing resources, while the latter represents the exploration of opportunities by integrating existing resources as a precondition. The patchwork of entrepreneurship was formally proposed by Baker and Nelson (2005) whose study found that “creating something out of nothing” was the most effective means of breaking the constraints of resource shortages for new firms; furthermore, the new value it creates is unique. Because new firms have inherent weaknesses, the inflows of external resources are limited. Consequently, it is both a challenge and an opportunity for new firms to selectively combine fragmented and neglected resources, with some resources mistakenly being considered worthless. Senyard et al. (2009) also noted that the patchwork of entrepreneurship has a significant role in promoting the financial and growth performance of new businesses. However, at the same time, the important role should not be overlooked of resource integration for internal integration (Cheng and Huizingh, 2014; Ma and Huang, 2016). Under the guidance of resource-based theory, Hitt et al. (2003) clearly emphasized that, as a driving force for developing opportunity, resource integration can promote strategic flexibility and performance in a new enterprise. Finally, the theory proposes the chain of resource integration–strategic flexibility–opportunity development–performance. Sirmon et al. (2011) and Teece (2007) recognized that resource integration, the ultimate method for simultaneously achieving internal consistency among resources and strategies, improves the ability to respond to environmental uncertainty and resolve the problems of facing ambiguous opportunities. Therefore, resource integration can cultivate a solid competitive advantage for enterprises. Clearly, when internal integration works, opportunities may be derived from resources, and the subsequent value of the opportunities depends on resources as well. Briefly, internal integration can promote the performance of new businesses.

*Hypothesis* 1: Internal integration is positively related to entrepreneurial performance.

Different from internal integration, external integration includes two other subdimensions: the combination of opportunity and resource identification and that of opportunity utilization and resource acquisition. The core of the former lies in identifying opportunities in dynamic environments that include uncertain market demand, massive amounts of data, large industrial trends, unique resource structures, and business models. Identifying opportunities is one of the main aspects of external integration. In fact, from the perspective of scholars taking opportunities as the primary goal, identifying opportunities is the core of entrepreneurship. Alvarez and Busenitz (2001) pointed out that opportunity identification is a source of competitive advantage for new firms and a basic precondition for bringing innovative goods and services to a market. Furthermore, effective opportunity identification allows companies to gain unimaginable profits (Baron, 2006; Bakker and Shepherd, 2017). However, enterprises must pay attention to the value of resources in this process, especially the value of knowledge resources. Obtaining more opportunities, however, does not equate to owning more innovative opportunities, nor does it represent the opportunities that can create higher value (Zahra et al., 2005; Petti and Zhang, 2011). Only a higher level of prior knowledge can identify more valuable opportunities. Therefore, the integration of opportunity and resource identification has a positive effect on new firm performance. However, the relationship between opportunity identification and performance is not linear. In regard to the integration of opportunity utilization and resource acquisition, effective access to external resources – such as financing, loans, government policies, support from partners, partnerships with suppliers, and a good reputation from customers – can significantly promote new firm performance.

*Hypothesis* 2: External integration is positively related to entrepreneurial performance.

Finally, internal and external integration are not integral. Similar to the mutual promotion and restraint of the yin and yang, the opposition between the two growth models (internal and external integration) is not absolute. Therefore, “the cultivation of both internal and external” integration is crucial for start-ups (Ennen and Richter, 2010; Song et al., 2010). For internal integration, combining the piecing together of resources with the recognizing of opportunities develops new opportunities – creative ones – with extremely limited resources. However, for external integration, the combination of opportunity and resource identification leads to new discovery opportunities in an uncertain environment. An opportunity type can be easily overlooked within a single kind of growth model. Similarly, regarding the combination of assigning resources and utilizing opportunities, one model of internal integration, collecting internal resources, can affect the outcome of exploiting opportunities, certainly in terms of enriching resources and enhancing stability, but also by neglecting the role of new resources outside the firm. Two key external and institutional factors – especially in China, which suffer from a transitional economy – imply multiple hidden resources, which are frequently fatal to firm success (Samuelsson and Davidsson, 2009). Therefore, although internal and external integration are opposed, they promote each other. The IOR reflects a balanced and reciprocal mechanism between them, namely, they interact. Therefore, the study proposes the third hypothesis:

*Hypothesis* 3: The IOR is positively related to entrepreneurial performance.

### IOR, Interpersonal Emotion Regulation, and Entrepreneurial Performance

In recent years, an increasing number of researchers have paid attention to emotion research in the field of organizational behavior. Emotion plays an important role in individual work outputs, team interactions, organizational decision-making, and reforms. Emotion has an important impact on many processes and results to which managers pay attention, such as conflict, cooperation, creativity, etc. Therefore, this paper discusses the impact of the IOR on entrepreneurial performance from the perspective of emotional regulation. Since China’s economy is transitional, both the institution and market are highly uncertain. In this context, social integration contributes to the realization of the firm’s new idea of constructive relationships among group members – manifested in aspects, such as cohesion, collaboration, and interpersonal facilitation (West, 2002; Hulsheger et al., 2009) – as well as in the influence of shared feelings within the team on social integration (Keltner and Haidt, 1999). Because entrepreneurship is an interpersonal process, the organization is an aggregation of emotional resources based on motivation, learning, and change. When an organization uses emotional ability to integrate and guide its internal emotion (Huy, 1999), it not only needs to be supported by strategic tools, such as selection, representation, and training but also needs to strictly conform to the organizational value level to achieve consistency and matching between organizational emotion and strategic actions. Emotion, particularly positive emotion, is the driving force of employee performance (i.e., emotions with positive values) and can promote a range of performance-related behaviors in the workplace (Rothbard and Wilk, 2011). For roles such as those played by supervisors and mentors who are responsible for improving the task performance of others (e.g., team members and clients), strategically improving the positive emotion of others may be an important means of improving their task performance (Niven, 2016). One way to improve the positive emotion of others is to use interpersonal affective regulation, which is intended to consciously initiate, maintain, or modify the occurrence, intensity, or duration of other people’s emotions (Niven et al., 2009). In short, interpersonal emotion regulation is effective for start-ups who must deal with the uncertainty of China’s transitional economy.

Based on the emotion regulation process model theory proposed by Little et al. (2012), the interpersonal emotion measurement tool proposed by Holman and Niven (2019) divides the regulation strategies into four categories: cognitive reappraisal, attentional deployment, situation modification, and relational engagement. Cognitive reappraisal attempts to influence the emotions of others by expressing and changing their cognition. Attentional deployment intends to focus or divert attention away from troublesome problems. These two strategies focus on the positive emotions of others, which are summarized as positive emotion regulation in this paper. Situation modification is defined as changing the situation to modify the influence on the emotions of others. Relational engagement is more concerned with relationships with others. These two strategies belong to interpersonal relationship regulation. By disentangling the question of whether the synergistic effects of opportunities and resources on entrepreneurial performance are restricted by levels of interpersonal emotion regulation in developing markets, this work extends the entrepreneurial performance created by high levels of IOR.

According to feeling-as-information theory and broaden-and-build theory of positive emotion, positive emotion has two functions: expansion and construction. That is, positive emotion can expand the basic cognition, thinking, and action categories of individuals; promote individuals in breaking through established limits; and produce more valuable thoughts. In addition, positive emotion can promote individuals to build cognitive, social, and other resources and provide sustainable resources for their growth (Fredrickson, 2001). When enthusiasm and activity ability are improved, existing behavior habits will be improved accordingly so a series of performance-related behaviors can be improved (Fredrickson and Branigan, 2005). Especially when an organization is faced with creative tasks and important decisions, such as internal and external integration actions, positive emotion can enhance the cognitive flexibility of team members, motivate them to find more similarities or differences between things, connect and integrate different information (Isen, 2000), and quickly make more thorough and effective choices. Moreover, in terms of attention, positive emotion can expand the visual search pattern, especially in the visual domain of peripheral stimuli, and can update individuals’ mental representations of the environment (Friedman and Forster, 2010). By promoting the integration of new and the existing knowledge, positive emotion can help to establish a long-term task knowledge system and make it easier to generate and implement creative ideas, which can more effectively transform cognition into high-quality performance (Konradt et al., 2003). In the process of external integration, a variety of uncertain problems can easily be solved innovatively, and it is no longer difficult for enterprises to create new products and new things. This situation may result in continuous competition for resources for the organization and improve organizational performance. Therefore, positive emotion regulation can affect the significance level of the relationship between the IOR and entrepreneurial performance. Accordingly, we hypothesize the following.

*Hypothesis* 4: The IOR is more positively related to entrepreneurial performance when positive emotion regulation is higher.

The second strategy of interpersonal emotion regulation is interpersonal relationship regulation. Compared with directly regulating individuals’ positive emotion, this strategy forms a positive emotional tone and good interaction and cooperation within the entrepreneurial team by forming a good interpersonal relationship among team members (Knight and Eisenkraft, 2015), which has an important impact on internal and external integration and entrepreneurial performance. During contextual modification and relational engagement, leaders remove, modify, or change situations and focus on improving team relationships. For example, if employees are anxious about completing work within a given time, supervisors may change the situation by reducing the amount of work required for the task or reassigning some responsibilities to others. The transmission of interpersonal care helps employees reevaluate the event so they can change the situation to modify its emotional impact (Niven et al., 2012) and further strengthen the benign group interaction norms and identity. Team identity enhances team cohesion, willingness to cooperate, and trust and moderates the sense of a shared vision, making the team more flexible and adaptable (Langfred, 2007). Entrepreneurial team members are highly interdependent because they share a goal, and they identify and develop opportunities together. Enterprises with good interpersonal adjustment ability perform better than their competitors do. In a turbulent market, entrepreneurial team members form a highly interdependent relationship because they share a goal. With different abilities, good communication and cooperation can produce cooperation effects, and employees can together identify and develop opportunities and integrate internal and external resources. Whether times are good or bad, employees can fully trust and encourage each other. In addition, in the complex and changeable entrepreneurial environment, relying on trust, high-quality information, and tacit knowledge that is effectively transferred, team members can efficiently learn and rapidly improve their innovation ability. This process effectively integrates heterogeneous knowledge resources and contributes to the improvement of entrepreneurial performance. Therefore, interpersonal relationship regulation can enhance the integration of opportunities and resources and cause the two strategies to concurrently promote each other, thus improving entrepreneurial performance.

*Hypothesis* 5: IOR is more positively related to entrepreneurial performance when interpersonal relationship regulation is higher.

**Figure 2:**
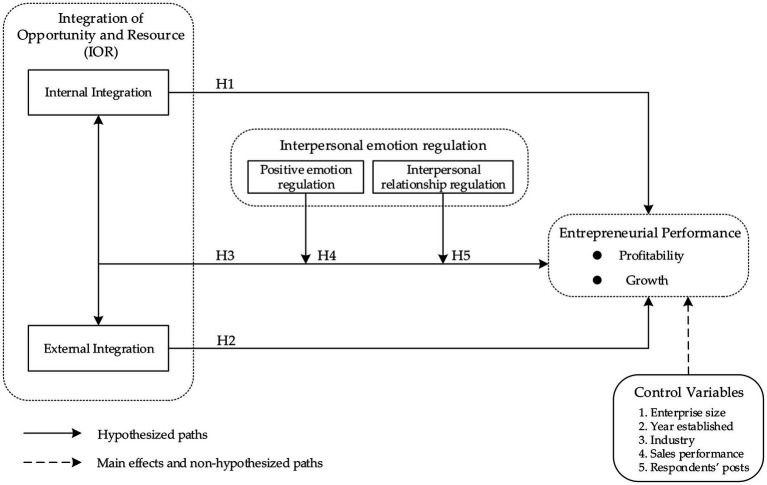
The research model.

Figure 2 presents the overall framework of this research.

## Methodology

### Data Gathering and Sample

In this study, to test the proposed hypotheses, we prepared a questionnaire that has not been published previously. Samples (291 firms) were identified from the China Small and Medium Enterprises Statistical Yearbook and the National Small and Medium-sized Enterprises Big Data Platform, both of which provide detailed information about medium-sized enterprises. Samples were then screened, and new enterprises of less than 8years old were used as the research objects. We invited senior and intermediate managers from alternative enterprises to be our informants using contact information from their companies’ websites because they receive global information about their company. This fact makes them valuable sources for evaluating each organization’s variables. To ensure that our sample can represent a larger population, first, the research areas included Beijing, Shanghai, Changchun, Shenyang, Hangzhou, and Shenzhen. These cities have different levels of development, including well-developed central cities and economically inactive cities in the north as well as southern coastal cities whose economies are thriving. Therefore, this study avoided the influence of regional economic development. Second, we chose informants who worked in start-ups of different industries when collecting data. Finally, we chose informants of different ages, ranging from 18 to 62years.

Simultaneously, we utilized different investigation methods, including email messages and face-to-face conversations but the same questionnaires to collect data. Before the investigation, we promised participants that the detailed information they provided about their companies would remain confidential. Additionally, we clarified in the questionnaires that we would continue the investigation only when the informant agreed. The data-collection process lasted for 10months, from November 2019 to September 2020. The data collection was mainly divided into three channels. First, after telephone appointments with managers, we collected 78 valid questionnaires through a survey of enterprises *via* face-to-face questionnaires completed by managers, without any invalid questionnaires. Second, focusing on the distribution of EMBA students, who were all middle or top managers, we collected 94 valid and six invalid questionnaires (those omitting fill-in items). Third, we distributed the questionnaires by email, collecting 113 questionnaires, excluding 11 invalid ones (which were completed in less than 5min), and retaining 102 high-quality ones. To ensure the questionnaire quality, we excluded those completed in less than 5min and those with missing items. Finally, 274 high-quality and effective questionnaires were collected. Third, the characteristics of responding businesses were compared to those of the nonresponding ones to reduce the possibility of nonresponse bias. We carried out a T test on the control variables, and the results for invalid and valid results indicated no significant difference between the respondents and the nonrespondents (refer Table 1).

**Table 1:** Characteristics of survey samples.

Enterprise size		Year established		Average turnover level in recent 3years		Age	
1–10 people	17.8	1year or less	7.1	Half a million or less	22.7	18–28	8.8
11–50 people	23.4			Half a million to 1million	11.1	29–39	48.5
51–100 people	13.8	1–3years	18.6	1–2million	5.9	40–50	24.8
101–300 people	19.3	3–5years	27.1	2–3million	7.1	51–61	10.2
More than 300 people	25.7	5–8years	47.2	3–5million	9.7	62 or above	7.7
				More than 5million	43.5		
Industry			Posts of respondents			Research area	
Software and communications	17.1	Transportation, storage and rent	13.7	State-owned	14.5	Shenyang	11.3
Manufacturing	17.8			Foreign		Changchun	31.0
Real estate	7.0	Transportation, storage and rent	5.9	investment	5.6	Beijing	15.0
Energy and environmental protection	2.9	Accommodation and catering	34.9	Private	70.2	Shenzhen	15.0
Finance	0.7			Joint venture	9.7	Hangzhou	13.5
						Shanghai	14.2

All figures in the table are percentages (%).

### Variable Measurements

Based on the hypotheses, the following section lists the required measurement items of this paper. We invited a professional scholar, an industry entrepreneur, and a representative from a government department to test the questionnaires to guarantee the scientific nature of these issues. We made some minor adjustments based on their recommendations to avoid misunderstanding the respondents. All the items were measured at five levels, where “1” represented a very insignificant description of the actual situation of the respondents and “5” was extremely consistent.

The IOR was the independent variable, which was reflected by the synergies between internal and external integration. Internal integration includes the integration of opportunity creation and resource patchwork and that of opportunity utilization and resource matching. We used four items that principally come from Baker and Nelson (2005) and Alvarez and Busenitz (2001) to measure the former and five items coming from Sarasvathy (2009) to measure the latter. External integration includes the integration of opportunity discovery and resource identification and that of opportunity utilization and resource acquisition. The three measurements of the former were mainly based on the research of Casson and Wadeson (2007) and Shepherd and Detienne (2005). The other five measurements of the latter were mainly based on the research of Timmons et al. (1994). Interpersonal emotion regulation, which consisted of positive emotion and interpersonal relationship regulation, was the mediating variable. The study utilized six items that principally came from Holman and Niven (2019) to measure interpersonal emotion regulation. The dependent variable was entrepreneurial performance, which includes profitability and growth. Because the scale measuring entrepreneurial performance is well developed, this study used six items to measure entrepreneurial performance. This paper applied as control variables enterprise size, the year the firm was established, the industry to which the company belongs, the average turnover level in the past 3years and the respondent posts pertaining to the enterprise (Zahra, 2012).

### Reliability Test and Factor Analysis

Table 2 displays the reliability and validity of the test results regarding internal and external integration, interpersonal emotion regulation, and entrepreneurial performance. We employed Cronbach’s *α* coefficient tests on the reliability of the scale and applied the variance contribution rate of the first principal component to test the scale validity. Better validity generally requires a variance contribution rate of 60%. The *α* coefficient of each variable was greater than 0.85, which was an acceptable level of reliability. The variance contribution rate of the first principal component of each variable was higher than 70%. Moreover, the factor loads of most items were greater than 0.8, revealing relatively high validity. Therefore, the scale employed in the study was reliable and valid (refer to Table 2).

**Table 2:** Results of the reliability and validity test.

Items				Factor load	*α* coefficient	Variance contribution rate/%
IOR	Internal integration	Integration of opportunity-creation and resource-patchwork	We can often develop new products or services with the limited knowledge and technology we have at hand.	0.841	0.907	73.179
			We are often able to invest and develop new projects based on the limited funds or channels available to us.	0.829		
			We often get some new ideas or ideas based on our existing teams and members.	0.853		
			We can often create some new modes of cooperation or value on the basis of our existing partnership.	0.879		
		Integration of opportunity-utilization and resource-matching	We can often make use of existing relationship channels to create new business opportunities that are more valuable.	0.874	0.907	78.289
			In the process of achieving business opportunities, we can adjust and configure the existing resources in a gradual way	0.883		
			In the process of achieving business opportunities, we can give full play to the complementarity of resources, such as the complementary skills of the members, team collaboration, etc.	0.899		
			In the process of achieving business opportunities, we can optimize the allocation of scarce resources (scarce patents, talents, etc.).	0.884		
			In the process of achieving business opportunities, we can make use of free resources to maximize the utility of existing resources.	0.873		
	External integration	Integration of opportunity-discovery and resource-identification	We can often identify new business opportunities by identifying new information, intelligence, etc.	0.887	0.930	78.409
			We can often identify new business opportunities by identifying new technological trends or service patterns outside the enterprise.	0.913		
			We can often identify new business opportunities by identifying potential customer needs from the external market.	0.902		
			We can often identify new business opportunities by identifying action trends of industry leaders.	0.887		
			We can often identify new business opportunities by identifying changes in the industry’s raw materials, tax benefits, etc.	0.837		
		Integration of opportunity- utilization and resource- matching	We often use bank loans, such as debt financing, to achieve business opportunities.	0.904	0.938	80.334
			We often use equity financing means such as venture capital to achieve business opportunities.	0.903		
			We often achieve business opportunities by introducing new patent technology and talent.	0.886		
			We often use the government’s help to achieve business opportunities.	0.889		
			We often develop new strategic partnerships to achieve business opportunities.	0.899		
Interpersonal emotion regulation	Positive emotion regulation		My supervisor gave me helpful advice.	0.918	0.932	88.129
			My supervisor discussed my positive characteristics with me.	0.955		
			My supervisor made me laugh.	0.943		
	Interpersonal relationship regulation		My supervisor did something nice with me.	0.918	0.892	82.488
			My supervisor listened to my problems.	0.932		
			My supervisor spent time with me.	0.874		
Entrepreneurial Performance	Profitability		The main businesses of the company always keep a high market share.	0.911	0.909	84.693
			Corporate profit margins have been kept at a very good level.	0.926		
			The investment return of the company is leading in the industry.	0.923		
	Growth		Compared with other companies in the same industry, our employee numbers increase rapidly.	0.933	0.940	89.393
			Compared with other companies in the same industry, our sales grow faster.	0.956		
			Compared with other companies in the same industry, the scale of us increase rapidly.	0.948		

In order to verify whether the distribution of variables conforms to the normality, this paper also uses the method of normality moment to test the skewness and kurtosis of IOR, interpersonal emotion regulation, and entrepreneurial performance. The results show that the skewness coefficient of IOR is 0.250 and the kurtosis coefficient is 0.383. The skewness coefficient of interpersonal emotion regulation is 0.078 and the kurtosis coefficient is −0.509. The skewness coefficient of entrepreneurial performance is 0.034 and the kurtosis coefficient is −0.15. The results show that the variables in this study correspond to normal distribution.

In addition, Harman’s single-factor test was used for the common method variance (CMV) test. A principal component analysis was conducted for all the questions in the sample questionnaire, and the table of “total variance explanation” was checked in the output results. The eigenvalue before rotation was greater than 1 as the judgment criterion. Furthermore, if the explanatory variation of the first factor is less than 50%, it is considered that there was no serious CMV (Fuller et al., 2016), and the test result of this study was 36.76%. However, considering the limitations of Harman’s single-factor test, this study introduced method factors to further test the CMV of the scale. Based on the CFA model in this study, the method factors were added as the overall factors to establish the two-factor model, and the change was observed in the fitting degree of the overall model (Podsakoff et al., 2003; Williams and McGonagle, 2016). AMOS software was used for the calculations to determine that after adding the method factors to the original CFA model, the CFI and TLI were increased by 0.092 and 0.096 (both less than 0.1), respectively, and the RMSEA was reduced by 0.029 (less than 0.05). The model fitting index did not improve significantly, indicating that there was no serious common method deviation.

## Results

### Descriptive Statistics and Correlation Coefficients

Table 3 delineates the descriptive statistical results of each variable and the correlation coefficient matrix. As shown in Table 3, the mean value of the variable varied from 2 to 4, and the SD was relatively low, which indicated that the fluctuation range of the variable was within a reasonable range. Internal and external integration were positively correlated with entrepreneurial performance, and positive emotion and interpersonal relationship regulation were positively related to entrepreneurial performance, which was consistent with the logic of the theoretical hypotheses. In addition, the enterprise size, firm establishment year, firm industry, sales performance level in the most recent 3years, and respondents’ posts were used as the five control variables in the test model. Direct and indirect interferences with the dependent variable were effectively avoided by controlling for these variables.

**Table 3:** Results of descriptive statistics and correlation coefficients.

S. no	Variable	Mean value	*SD*	1	2	3	4	5	6	7	8	9	10	11	12	13
1.	Integration of opportunity-creation and resource-patchwork	17.671	4.184													
2.	Integration of opportunity-utilization and resource-matching	14.237	3.122	0.716^**^												
3.	Integration of opportunity-discoveryand resource-identification	18.175	3.902	0.518^**^	0.544^**^											
4.	Integration of opportunity-utilization and resource-matching	16.689	5.030	0.391^**^	0.459^**^	0.715^**^										
5.	Positive emotion regulation	0.000	0.999	−0.091	−0.038	−0.081	−0.022									
6.	Interpersonal relationship regulation	0.000	1.000	−0.014	0.097	−0.004	0.011	0.585^**^								
7.	Profitability	0.000	1.000	0.662^**^	0.737^**^	0.561^**^	0.534^**^	−0.264^**^	−0.134^*^							
8.	Growth	0.000	1.000	0.626^**^	0.712^*^^*^	0.602^**^	0.574^**^	−0.173^**^	−0.092	0.895^**^						
9.	Enterprise size	3.109	1.478	0.260^**^	0.229^**^	0.214^**^	0.238^**^	−0.074	0.002	0.343^**^	0.295^**^					
10.	Year established	3.762	1.390	0.143^*^	0.183^**^	0.068	0.143^*^	0.004	0.023	0.236^**^	0.151*	0.624^**^				
11.	Industry	4.945	2.865	−0.275^*^^*^	−0.187^**^	−0.144^*^	−0.106	0.022	−0.009	−0.167^**^	−0.160^**^	−0.293^**^	−0.235^*^^*^			
12.	Sales performance	3.989	2.104	0.243^**^	0.243^**^	0.240^**^	0.264^**^	−0.026	0.009	0.335^**^	0.291^**^	0.789^**^	0.692^**^	−0.250^**^		
13.	Respondents’ posts	2.740	0.826	−0.085	−0.152^*^	−0.139^*^	−0.155^*^	0.076	0.028	−0.126^*^	−0.104	−0.180^**^	−0.140^*^	−0.045	−0.117	1

The sample number is 274.

* *p*<0.05;

** *p*<0.01.

### Multivariate Linear Regression Model and Results

Table 4 lists the results of the four multivariate linear regression models of the combination of internal and external integration, interpersonal emotion regulation, and entrepreneurial performance. The variance inflation factor (VIF) of each regression model was less than 10, which indicated that the method avoids multicollinearity. Model 1 was the benchmark model that tested the relationship between the control variables and the dependent variable (entrepreneurial performance). Model 1 indicated that enterprise size was significantly positively related to entrepreneurial performance, indicating that a larger enterprise results in higher entrepreneurial performance. However, the firm establishment year was positively correlated with entrepreneurial performance, suggesting that older companies had lower entrepreneurial performance. Model 2 examined the pathway pertaining to the influence of internal and external integration and positive emotion and interpersonal relationship regulation on entrepreneurial performance. The regression results of Model 2 showed that internal and external integration and interpersonal relationship regulation were significantly correlated with entrepreneurial performance. In this model, a one-point SD increase in internal integration caused a 58.8% change in profitability and a 52.2% change in growth. Furthermore, a one-point SD increase in external integration caused a 23.8% change in profitability and a 32.9% change in growth. This result emphasized that enterprises employing as their strategy internal or external integration secured better entrepreneurial performance and acquired the full advantages of interpersonal relationship regulation. The interactions between the respective variables were illustrated in Model 3. Model 4 revealed a positive interaction between internal and external integration and entrepreneurial performance, thus verifying Hypotheses 1 and 2. Compared to the results of Model 2, the combination of internal and external integration led to higher entrepreneurial performance, which validated Hypothesis 3. In Equations (1) and (2), a one-point SD increase in internal integration caused a 55.1% change in profitability and a 57.8% change in growth. Furthermore, a one-point SD increase in external integration caused a 40.1% change in profitability and a 63% change in growth. Compared with the results in Models 2 and 3, Model 4 contained the synergies of the combination of internal and external integration and positive emotion and interpersonal relationship regulation; consequently, the performance was higher. Therefore, internal and external integration and positive emotion regulation were significantly positively correlated with entrepreneurial performance, proving that excellent positive emotion regulation further promoted the positive synergies of the integration of internal and external integration on entrepreneurial performance. In addition, their internal and external integration, interpersonal relationship regulation, and entrepreneurial performance were positively correlated illustrating that preeminent interpersonal relationship regulation further improved the significant effect of the combination of internal and external integration on entrepreneurial performance. Therefore, Hypotheses 4 and 5 were verified.

**Table 4:** Results of the relationship between IOR, interpersonal emotion regulation and entrepreneurial performance.

	Dependent variable: Entrepreneurial performance								Results
	Profitability				Growth				
	Model 1	Model 2	Model 3	Model 4	Model 1	Model 2	Model 3	Model 4	
Control paths									
Enterprise size	0.119^*^	0.056	0.048	0.059	0.110^*^	0.056	0.044	0.048	
Year established	−0.026	0.041	0.034	0.008	−0.099^*^	−0.028	−0.033	−0.064^*^	
Industry	−0.027	0.022^*^	0.017	0.009	−0.031	0.015	0.011	0.004	
Sales performance	0.091^*^	0.012	0.010	0.005	0.107^**^	0.018	0.019	0.020	
Respondents’ posts	−0.096	0.033	−0.000	−0.017	−0.086	0.044	0.018	0.001	
Main effect paths									
Internal integration		0.588_*_^**^	0.484^***^	0.332^***^		0.522^***^	0.442^***^	0.277^***^	
External integration		0.238^***^	0.250^***^	0.307^***^		0.329^***^	0.365^***^	0.430^***^	
Positive emotion regulation		−0.170^***^	−0.115^**^	−0.112^**^		−0.073	−0.043	−0.101^*^	
Interpersonal relationship regulation		−0.059	−0.012	0.012		−0.069	−0.048	0.022	
Two-way interaction paths									
Positive emotion regulation × interpersonal relationship regulation			0.061	0.118^***^			0.033	0.101^**^	
Internal integration × positive emotion regulation			0.160^***^	0.060			0.213^***^	0.076	
Internal integration × interpersonal relationship regulation			−0.047	−0.095			−0.078	−0.142^**^	
External integration × positive emotion regulation			−0.173^***^	−0.180^***^			−0.224^***^	−0.214^***^	
External integration × interpersonal relationship regulation			−0.036	−0.034			0.057	0.047	
Hypothesized paths									
H1: Internal integration				0.332^***^				0.277^***^	Support
H2: External integration				0.307^***^				0.430^***^	Support
H3: Internal integration × external integration				0.149^***^				0.160^***^	Support
H4: Internal integration × external integration × positive emotion regulation				0.063^**^				0.139^***^	Support
H5: Internal integration × external integration ×interpersonal relationship regulation				0.096^***^				0.068^**^	Support
N	274	274	274	274	274	274	274	274	
R^2^	0.139	0.669	0.696	0.737	0.115	0.619	0.643	0.701	
Adjusted R^2^	0.123	0.658	0.679	0.719	0.098	0.606	0.623	0.682	
△R^2^	−	0.535	0.021	0.040	−	0.508	0.017	0.059	
*F* value	8.707	59.541	42.419	42.220	6.967	47.847	33.342	35.461	

* *p*<0.10;

** *p*<0.05;

*** *p*<0.01.

These analyses indicated that the empirical results supported the aforementioned theoretical expectations, and Hypotheses 1–5 were verified. The combination of internal and external integration promoted entrepreneurial performance, and interpersonal emotion regulation played a positive role (refer to Table 4).

## Discussion

**Figure 3:**
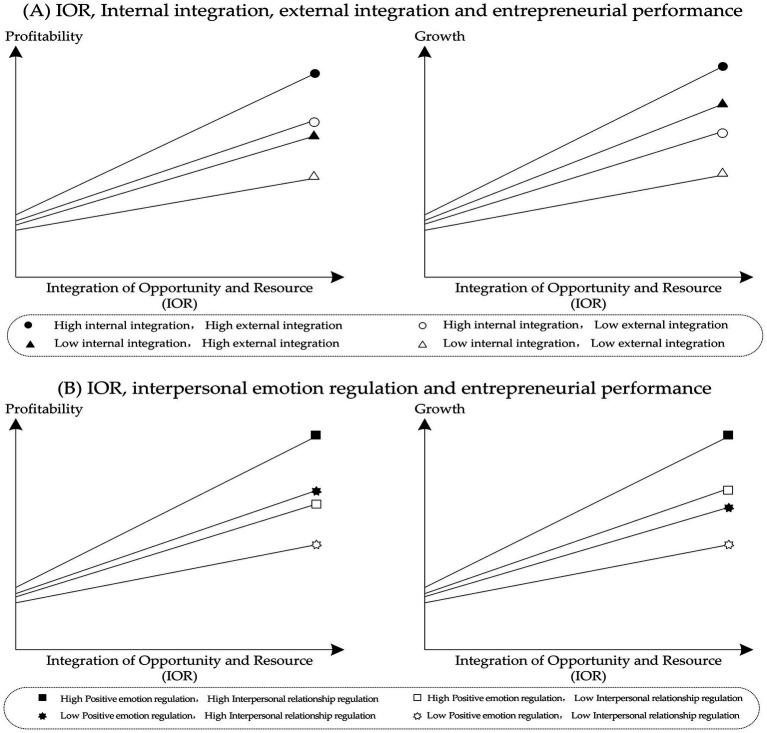
Plots of the interaction effects.

Since the entrepreneurship literature has focused on the matching of opportunities and resources, there has been a gap in their integration. Neither opportunity- nor resource-oriented theory breaks through the limitation of a single perspective, and studies from the integration perspective are very rare. Based on the existing theoretical gap, for the first time, we regard opportunities and resources from a holistic perspective and propose the concept of the integration of opportunities and resources (IOR). On this basis, the research explored the relationship among the IOR, interpersonal emotion regulation, and entrepreneurial performance, and all results are shown in Figure 3. In this context, our study contributes to both theory and practice.

### Theoretical Implications

First, the study overcomes the limitations of the unitary perspective of the dualistic theory of entrepreneurship, whose purpose is exploring the synergies of opportunities and resources, revealing the relationship between them. The application of system theory can not only help to reveal the main context of entrepreneurship, and but also can transform the entire discipline of entrepreneurship into a more scientific category. Therefore, the proposal of system theory provides a new research perspective for entrepreneurship. The system composed of opportunities and resources can deal with uncertain risks more stably in entrepreneurial research, and promote entrepreneurial performance more stably and comprehensively. The interaction and integration between opportunities and resources is a systematic process, which is complex, dynamic, multi-level, and multi-dimensional. IOR is the core and driving force of entrepreneurship.

Second, the study shows a complementary effect between internal and external integration, which are not independent concepts. Entrepreneurial performance will be higher when enterprises engage in IOR behavior. Creating opportunities is a precondition of internal integration, which unifies of opportunities and resources by piecing together and assigning internal resources. Internal integration focuses on the entrepreneurial process within an organization and advocates that opportunities are created internally and their value can be maximized by exploiting the limited resources at hand. Taking as a prerequisite opportunity discovery, external integration develops opportunities by identifying and acquiring external resources. External integration focuses on aspects outside the enterprise, believing opportunities are derived from external sources, including access to finance, government support, the identification of underlying information, technology trends, etc. External integration consists of the interaction between the identification of external resources and the discovery of opportunities. However, internal and external integration have their own limitations. In internal integration, piecing together resources will lead to opportunity limitations and detachment from reality, although it can break through the restrictions of resource constraints. Internal integration needs external integration to compensate for shortages. In external integration, enterprises identify external resources to discover and exploit potential opportunities by acquiring resources. The results deepen the understanding of different entrepreneurial strategic directions for subsequent research.

Third, the findings show that positive emotion and interpersonal relationship regulation further maximize the performance associated with internal and external integration. In a transitional economy, start-ups must flexibly utilize internal and external integration. In the integration process, leaders should focus not only on overcoming the uncertainty created by the environment but also on coordinating the team relationship and members’ emotions to make the entrepreneurial activities proceed smoothly. Interpersonal emotion regulation is an important psychological function in social behavior that aims to stimulate, change, or regulate the emotions of others, such as organization members. However, this function is still poorly explored in organizational contexts, so our article aims to gain insights into how the interpersonal emotion regulation can be a tool for leaders to influence performance in the context of internal and external integration in organizations. This effort is consistent with previous work on the role of emotional regulation in leadership and organizations (Riggio and Reichard, 2008; Madrid et al., 2019; Torrence and Connelly, 2019), expanding the application of basic psychology in the organizational field (Gross, 2013; Niven, 2017). The level of interpersonal emotion regulation becomes essential for explaining the relationship between strategy orientation and performance.

### Managerial Implications

First, this paper explores the synergy and relationship between opportunities and resources. Doing so provides a clear solution for start-ups on how to coordinate opportunities and resources in a dynamic environment, which is critical to improving corporate performance. Firms identifying opportunities in the market will inevitably be followed by a multitude of competitors, which will decrease the value of opportunities and make market competition increasingly fierce. Hence, a shortage of resources will again be an enterprise limitation; however, internal integration will be helpful in solving this problem. External integration needs internal integration to compensate for the shortage of resources caused by fierce market competition. In summary, the IOR represents a high level of integration between internal and external integration, each of which can compensate for the other’s deficiencies to achieve high enterprise performance in a transitional economy. Therefore, in transitional economic environments, the strategic positioning and implementation must be carried out from the internal and external aspects of the enterprise, and companies need to coordinate and use different strategies and capabilities, such as internal and external integration. Different entrepreneurial strategies should not be regarded as contrary but as complementary.

Second, the results show that interpersonal emotion regulation plays an important role in the relationship between the IOR and entrepreneurial performance, this conclusion provides more comprehensive guidance for managers to predict and influence employee behavior. Start-ups need to effectively regulate interpersonal emotion in a turbulent system and dynamic competitive market to achieve higher returns. Therefore, entrepreneurs should first realize that interpersonal emotional regulation plays an important role. And then we encourage start-ups to engage in interpersonal emotion regulation activities. This step requires enterprises to pay attention to the training of identifying leaders’ tendencies and abilities in interpersonal emotion regulation from the perspective of leaders’ development plans and to improve and construct enterprise effective strategies. Specifically, a leader’s development plan should include assessment and training of interpersonal emotional regulation and emotional intelligence skills, for example, through role-playing and simulation. Alternatively, when recruiting candidates for team leadership positions, the personnel selection system could use interpersonal emotional regulation as an evaluation variable.

### Research Limitations and Future Research Directions

The paper has two limitations. First, our data source is singular, and the transitional economy in China is unique. Other developing countries may have more diverse background elements. For example, cultural factors in different countries (such as collectivism and performance orientation) and informal institutional constraints (such as levels of political steadiness, putrefaction, judicial fairness, availability of education, and other common resources) may have different effects on strategic directions and business success. Future research should augment this area. Second, we selected entrepreneurial companies established within the last 8years as the sample to ensure the consistency of the data. However, IOR and interpersonal emotion regulation are not negligible for mature enterprises. Therefore, studying the relationships among variables with combined life cycles can lead to conclusions that are more universal.

In addition, the results are consistent with institutional principles. The results emphasize that interpersonal emotion regulation (the core element in the development of a transitional economy in China) helps firms to effectively collect knowledge, acquire imperative channels, and develop new business networks under information asymmetries. Finally, this study suggests that entrepreneurial companies in transitional economic contexts should have stronger capabilities to develop the IOR and conduct interpersonal emotion regulation activities. However, the acquisition of these strategic capabilities is not simple but expends countless time and manifold resources. Therefore, follow-up research should help companies overcome these barriers, including through the construction of government protection agencies to enable start-ups to implement the best entrepreneurial practices in the commercial market group.

## Data Availability Statement

The raw data supporting the conclusions of this article will be made available by the authors, without undue reservation.

## Ethics Statement

Ethical review and approval was not required for the study on human participants in accordance with the local legislation and institutional requirements. Written informed consent for participation was not required for this study in accordance with the national legislation and the institutional requirements.

## Author Contributions

NL and YS: writing. DJ: processing data. XY: revising. All authors contributed to the article and approved the submitted version.

## Funding

This work was supported by the Jilin Province Social Science Foundation Project (2020C054) and Guangxi Science and Technology Plan Project (Guangxi Science and Technology Base and Talent Special Project: AD20159069).

## Conflict of Interest

The authors declare that the research was conducted in the absence of any commercial or financial relationships that could be construed as a potential conflict of interest.

## Publisher’s Note

All claims expressed in this article are solely those of the authors and do not necessarily represent those of their affiliated organizations, or those of the publisher, the editors and the reviewers. Any product that may be evaluated in this article, or claim that may be made by its manufacturer, is not guaranteed or endorsed by the publisher.
